# The Effects of Exercise Training in Addition to Energy Restriction on Functional Capacities and Body Composition in Obese Adults during Weight Loss: A Systematic Review

**DOI:** 10.1371/journal.pone.0081692

**Published:** 2013-11-25

**Authors:** Clint T. Miller, Steve F. Fraser, Itamar Levinger, Nora E. Straznicky, John B. Dixon, John Reynolds, Steve E. Selig

**Affiliations:** 1 Centre for Exercise and Sports Science, School of Exercise & Nutrition Sciences, Deakin University, Melbourne, Australia; 2 Institute for Sport, Exercise and Active Living (ISEAL), School of Sport and Exercise Science, Victoria University, Melbourne, Australia; 3 Human Neurotransmitters Laboratory, Baker IDI Heart & Diabetes Institute, Melbourne, Victoria, Australia; 4 Clinical Obesity Research Laboratory, Baker IDI Heart and Diabetes Institute, Melbourne, Australia; 5 Biostatistics Unit, Faculty of Health, Deakin University, Melbourne, Australia; University of Buenos Aires, Cardiovascular Pathophysiology Institute, Argentina

## Abstract

**Background:**

Obesity is associated with impairments of physical function, cardiovascular fitness, muscle strength and the capacity to perform activities of daily living. This review examines the specific effects of exercise training in relation to body composition and physical function demonstrated by changes in cardiovascular fitness, and muscle strength when obese adults undergo energy restriction.

**Methods:**

Electronic databases were searched for randomised controlled trials comparing energy restriction plus exercise training to energy restriction alone. Studies published to May 2013 were included if they used multi-component methods for analysing body composition and assessed measures of fitness in obese adults.

**Results:**

Fourteen RCTs met the inclusion criteria. Heterogeneity of study characteristics prevented meta-analysis. Energy restriction plus exercise training was more effective than energy restriction alone for improving cardiovascular fitness, muscle strength, and increasing fat mass loss and preserving lean body mass, depending on the type of exercise training.

**Conclusion:**

Adding exercise training to energy restriction for obese middle-aged and older individuals results in favourable changes to fitness and body composition. Whilst weight loss should be encouraged for obese individuals, exercise training should be included in lifestyle interventions as it offers additional benefits.

## Introduction

Increasing obesity and central adiposity leads to a greater risk of developing obesity-related morbidities and disabilities [1], [2]. Diabetes mellitus, cardiovascular disease, sleep apnoea, dyspnoea, mental illness, osteoarthritis, foot and ankle tendinitis, plantar fasciitis, low back pain and chronic lower extremity pain all impact negatively on an individual's capacity to perform activities of daily living and are more prevalent in obese individuals [3], [4], [5], [6]. Further, reduced capacity for activities of daily living may occur prior to the development of these conditions and may be related to adverse metabolic and biomechanical changes associated with obesity [7], [8]. Obese individuals often experience the vicious cycle of low exercise capacity, physical disability and breathlessness leading to physical inactivity, in turn leading to further weight gain [9] and loss of physical function [10], [11]. In addition, midlife obesity alone can lead to a 5 times greater risk of developing old age frailty compared with healthy weight peers [12]. There appears to be an additive effect of obesity and low strength for the development of mobility disability and walking speed during aging. One recent study [13] observed a 17% decline in walking speed after six years in obese participants with low strength compared with 2% decline for non-obese, non-low strength group. Low strength was shown to have the greatest influence for decline in walking speed over time.

A lack of adequate lower extremity muscle strength and power in obesity may impair simple tasks such as walking and stair climbing [8]. Obese individuals may possess low cardiorespiratory fitness, strength and endurance relative to their body mass, reduced spinal flexibility and joint ranges of motion. All of these can increase the risk of pain and injury at work, increased absenteeism and reduced work capacity [14], [15], [16], [17]. At home, low relative physical condition associated with obesity may impair the capacity to undertake household chores, general activities of daily living and interfere with the ability to engage in social activities [18]. Low cardiorespiratory capacity and lack of regular involvement in exercise may reduce concentration, the capacity to deal with anxiety and stress, and impair cognitive function [19], [20], [21]. Regular aerobic or resistance exercise training influences physical fitness and functional capacity through improvements in muscular strength, power, endurance, and cardiorespiratory and vascular fitness. Aerobic exercise training specifically facilitates the improvement in central and peripheral cardiorespiratory, vascular and metabolic function, while resistance training improves muscular endurance, strength, power and hypertrophy [22]. The addition of specific exercise training to energy restriction in obesity may, in addition to changes in physical fitness confer favourable body composition outcomes [23].

Although the mechanisms that lead to weight gain or loss are complex and not fully understood (behavioural, environmental, inherited, and physiological) [1], [24], [25], [26], the final determination for alterations in weight can be viewed most broadly by an energy imbalance via one or both of energy over-consumption and low energy expenditure. Energy restriction and regular exercise training can independently influence the degree of fat mass loss and lean mass loss in overweight and obese individuals during weight loss interventions [27]. The implications for excessive lean mass loss during weight loss are well established. Lean mass is integral to the long term maintenance of metabolic rate, core body temperature, skeletal integrity, muscle strength, functional capacities [28], [29], and the prevention of sarcopenic obesity later in life [30]. During weight loss a greater proportion of lean mass is lost compared to when weight is regained [31], [32]. The resulting lean mass deficit and continued lack of physical activity during ageing may lead to increased risk of physical disability later in life [33], [34].

Rapid and substantial weight loss observed following bariatric surgery can produce immediate improvements in functional capacity in the absence of exercise training [35]. In older adults, it appears that engaging in relatively small amounts of physical activity such as regular incidental activity can reduce the risk of developing impaired physical function [36], [37], however a blunted response to exercise training can be seen in obese individuals exposed to the same training stimulus as their healthy weight peers [37]. In healthy populations, changes in strength and aerobic fitness can be directly related to the training stimulus [38] and the exercise training variables manipulated such as frequency, intensity, time or type of activity [22], [39], [40] There are no reviews that compare changes in functional capacity or fitness in obese adults following energy restriction with or without exercise training.

Currently, multicomponent methods such as DXA (Dual-energy X-ray absorptiometry) and MRI (magnetic resonance imaging) are preferred methods of measuring body composition for changes in fat free mass during weight loss as it reduces the reliance on algorithmic assumptions of fat free mass hydration and density which may be inaccurate for obese individuals [41], [42], [43]. Multicomponent body composition methods are more sensitive to changes in fat free mass compared with two-component models such as hydrodensitometry, air displacement plethysmography, bioelectrical impedance, near-infrared or skin folds [28], [44], [45] which may be more appropriate for those studies that result in small changes in weight.

Previous systematic reviews have compared changes in body composition using a variety of two-compartment body composition techniques but none have included objective measures of physical function or fitness following energy restriction with or without exercise training in obese adults. Weinheimer et al. [23] published on the effects of exercise training on fat free mass in middle aged and older adults. The primary outcome measure was body composition in older adults at increased risk of developing sarcopenic obesity. The study found that regular exercise training in addition to energy restriction may attenuate the loss of fat free mass in older adults compared to energy restriction alone. This follows a similar finding in an earlier review of overweight and mildly obese adults [46].Both reviews included two-compartment body composition techniques that estimate fat-free mass according to general assumptions of total body water and bone mineral density; but also may not be sufficiently sensitive to detect small changes in fat-free mass during weight loss [28], [47]. A third review [48] limited the inclusion of papers to those that employed the use of MRI and computed tomography to determine visceral adipose tissue changes following either aerobic, progressive resistance training or combined treatment in adults. This review showed that aerobic but not progressive resistance training or combined treatments were shown to lower visceral adipose tissue to a greater extent than control groups. To our knowledge there has not been a systematic review on changes in physical function, fitness and body composition in obese adults during energy restriction alone compared to energy restriction combined with exercise training. The importance of muscle quality (force per unit of cross sectional muscle area) is raised in one review but fitness or strength measures were not outcome measures reported in that review [23].

Body composition and measures of aerobic and muscular fitness may influence physical function. A decline in physical fitness and adverse body composition changes can occur during weight loss and during weight cycling which may increase the risk of developing reduced functional capacity and physical disability later in life. The purpose of this review is to examine the effects of exercise training when added to energy restriction for changes in body composition and cardiovascular and muscular fitness measures in obese adults.

## Materials and Methods

### Search strategy

**Figure 1:**
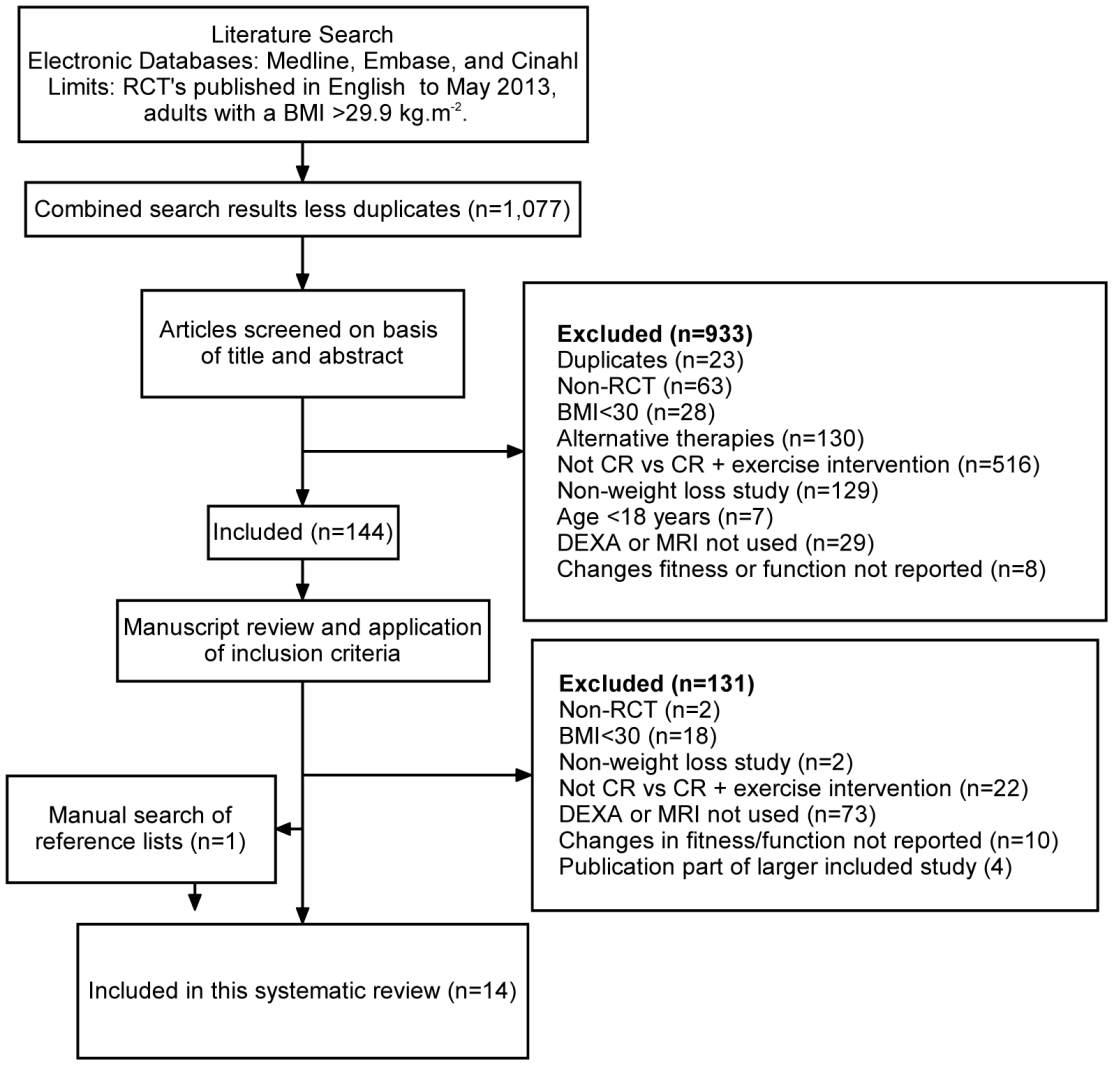
Method for selecting studies to include in this review.

Medline, Embase and Cinahl electronic databases were searched (Figure 1) for randomised controlled trials (RCTs) up to May 2013 using the following search strategy: keyword and categorical searches were performed (i) Weight loss, or body composition (ii) diet* or “diet therapy” or “diet restriction” or “caloric restriction” or “calorie” or “bariatric surgery” or “gastric banding” or “vertical banded gastroplasty”; (iii) exerc* or “physical activity” or aerobic* or “resistance training” or fitness. Categories i-iii were also combined using “AND”, limited to humans, reported in English, and adults aged 18+ years. In addition, reference lists of publications meeting the inclusion criteria were manually searched to identify any relevant studies not found through electronic searches. Two authors CM and SS independently assessed the suitability of each study for inclusion. Disagreements were resolved by discussion between the two researchers.

### Inclusion and exclusion criteria

Only RCTs that met the following criteria were included in this review: (i) published in English, (ii) cohorts were adults aged 18 years and older, (iii) the same energy restriction intervention was used in the energy restriction only and, combined exercise training with energy restriction arms of the study, (iv) mean BMI of participants ≥ 30 kg.m^2^, (v) Dual-energy X-ray absorptiometry (DXA), or magnetic resonance imaging (MRI), (vi) a minimum of two groups comprising energy restriction only and energy restriction plus exercise training. Studies were excluded if they did not report changes in objective measures of physical function or fitness. Outcome measures for inclusion were fat free mass and fat mass, and cardio-respiratory fitness, muscle strength or muscular endurance measures. In the case of more than one publication arising from the same study or patient cohort, the largest study only was included. Unblinded appraisal of risk of publication bias of reports to be included in the review was performed independently by two authors (CM and SS). Publications were assessed against pre-defined quality features [49] (Table 1) to be included in the review and analysis. These included: the method of treatment assignment; control of selection bias after treatment assignment; blinding; and outcome assessment (if blinding was not possible). Pre-specified analyses for assessing risk of bias across studies were not completed due to a lack of identified publications.

**Table 1:** Checklist for appraising the quality of studies included.

Condition	(a)	(b)	(c)	(d)
**Method of treatment assignment**	Correct, blinded randomization method described OR randomized, double-blind method stated AND group similarity documented	Blinding and randomization stated but method not described OR suspect technique (e.g. allocation by drawing from an envelope)	Randomization claimed but not described and investigator not blinded	Randomization not mentioned
**Control of selection bias after treatment assignment**	Intention to treat analysis AND full follow-up	Intention to treat analysis AND: 15% loss to follow-up	Analysis by treatment received only OR no mention of withdrawals	Analysis by treatment received AND no mention of withdrawals OR more than 15% withdrawals/loss-to-follow-up/post-randomization exclusions
**Blinding**	Blinding of outcome assessor AND patient and care giver	Blinding of outcome assessor OR (patient AND care-giver)	Blinding not done	
**Outcome assessment (if blinding was not possible)**	All patients had standardized assessment	No standardized assessment OR not mentioned		

There are numerous methods used to assess body composition, all with varying degrees of reliability and validity. We chose to select only those studies that employed the use of DXA or full body MRI. The rationale for this was to eliminate methods that rely on algorithmic assumptions of fat free mass hydration and density which may be inaccurate for obese individuals [41], [42], [43]. The methods that we included are more sensitive to changes in fat free mass compared with two-component models such as hydrodensitometry, air displacement plethysmography, bioelectrical impedance, near-infrared or skin folds [28], [44], [45]. Bariatric surgery that involves malabsorption of nutrients were not included as they have been found to interfere with the levels of gastrointestinal hormone ghrelin which may influence release of growth hormone and affect lean mass [50], [51]. Gastric banding was the only surgical procedure to be included as part of this review as a form of energy restriction.

In view of the heterogeneity in study design, interventions, age of participants, medication use, cardio-metabolic disease status, and outcome measures we provide a descriptive review rather than a meta-analysis of results. Each of the tables in the results provides the actual means and SD for each of the measures as reported in the 14 papers. In addition for Table 4, we have presented VO_2peak_ in absolute terms (ml.min^−1^), and relative to total body mass (ml.min^−1^.kg BM^−1^) and lean body mass (ml.min^−1^.kg LM^−1^). We have used the means of total body mass or lean body mass, as appropriate for each study, to calculate VO_2peak_ from the absolute measure of VO_2peak_, or vice versa.

### Statistical methods for body mass and composition

Percentage changes in reported pre- and post- means of body mass and composition were calculated for groups treated in selected studies. Studies that reported skeletal mass rather than fat-free mass were excluded from these summaries. The published report by Daly et al [52] study was supplemented with a personal communication which enabled the percentage change in the means to be calculated. Data from reports were extracted independently and verified by CM and SS.

Predicted means of the percentage changes for each of the four types of treatment group (ER =  Energy Restriction, ER+A =  Energy Restriction plus Aerobic Training, ER+R =  Energy Restriction plus Resistance Training, and, ER+A+R =  Energy Restriction plus a combination of Aerobic and Resistance Training) were calculated by fitting a linear mixed model using residual (or restricted) maximum likelihood (REML). The treatments were regarded as fixed effects and the studies, and groups within studies, were regarded as random effects. Estimates of the standard deviations of the percentage changes in the means could not be recovered from all the published reports of the studies and so the mixed model analysis was unweighted. The F-test was used to test for significant variation between the four types of treatments and least significant difference (LSD) tests were used to explore differences between the groups. All tests were conducted at the 5% significance level. The REML directive in the GenStat statistical system (Release 14.2) was used to calculate the means and perform the statistical tests [53].

## Results

A total of 1,077 citations matching the search criteria were found in the initial literature search. The abstracts of these publications were screened and 933 articles were excluded as they did not meet the inclusion criteria (Figure 1). The full text of 144 articles were retrieved. A further 131 were excluded from these, leaving 13 RCTs. A manual search of the reference lists of the 13 articles that were included identified one additional paper and so was added to this review (Table 2). Two papers were identified that presented the same data from one group of participants [54], [55]. Changes in body mass and body composition were the primary and secondary outcome measures in one of these papers [55] and was therefore selected for inclusion.

**Table 2:** Characteristics of included studies.

Reference	Sex (n)	Mean age	Mean BMI	Study Duration	Completed	Primary outcomes	Energy restriction protocol	Exercise protocol	Exercise adherence
Villareal 2011 [59]	F (33) M (21)	70	37	6 mo.	87%	Physical performance score	500–750 kcal.day^−1^ deficit; 1g protein.kg bw.d^−1^	3×90 min.wk^−1^ supervised RT+AT	83 (80-88%)
Nicklas 2009 [64]	F (95)	58	33	5 mo.	85%	Abdominal adipose tissue, cardiovascular disease risk factors	400 kcal.day^−1^ deficit; *50–60/15–20/25–30	**ME**: 3×55 min.wk^-1^ supervised AT; **VE**: 3×30 min.wk^-1^ supervised AT	**ME**: 92.6% (±5.5); **VE**: 90.0% (±8.7)
Toledo 2008 [67]	F (10) M (6)	44	34	5 mo.	100%	Mitochondrial capacity	∼25% energy deficit.day^-1^	3–5×40 min.wk^-1^ AT; ≥1 session supervised	1.5 (0.2 days) Supervised; 2.5 (0.4 days) Unsupervised
Straznicky 2010 [65]	F (20) M (20)	55	32	3 mo.	92%	Sympathoinhibition, metabolic syndrome components	600 kcal.day^-1^ deficit; *48/22/30	3–4×40 min.wk^-1^ AT; 1 session supervised	NR
Amati 2008 [60]	F (15) M (13)	67	32	4 mo.	100%	Exercise efficiency and economy	^a^500–1,000 kcal.day^-1^ deficit; <30% cal from fat	3–5×45 min.wk^-1^ AT; 3 sessions supervised; 2 sessions unsupervised	3.6 (0.2 days)
Messier 2010 [63]	F (107)	58	32	6 mo.	78%	Psychosocial factors	500–800 kcal.day^-1^ deficit; *55/15/30	3×(2–4 sets of 6 exercises).wk^-1^ supervised RT	NR
Frimel 2008 [62]	F (18) M (12)	70	37	6 mo.	97%	Fat-free mass	750 kcal.day^-1^ deficit; *50/20/30	3×90 min.wk^-1^ supervised RT+AT	100%
Daly 2005 [52]	F (13) M (16)	67	32	6 mo.	100%	Bone mineral density, lean mass	ER to induce 0.25kg wt loss.wk^-1^<30% kcal from fat	3×(3 sets x 8–10 reps of 9 exercises).wk^-1^ supervised RT	88%
Wycherley 2010 [66]	F+M (59)	51	35	4 mo.	71%	Body mass, body composition, cardiovascular disease risk factors	1,400–1,600 kcal.day^-1^; *43/33/22 (HP) *53/19/26 (STD)	3×(2×8–12 of 8 exercises).wk^-1^ supervised RT	93 (±8.5%)
Bouchard 2009 [61]	F (23)	62	32	3 mo.	96%	Physical capacity	ER to induce 0.5–1.0kg wt loss.wk^-1^; *55/15/30	3×(3 set x 8 reps of 9 exercises). wk^-1^ supervised RT	NR
Janssen 1999 [57]	F (30) M (30)	41	33	4 mo.	100%	Skeletal muscle mass	1,000 kcal.day^-1^ deficit; <30% kcal from fat.	AT: 5×34 min.wk^-1^ supervised; RT: 3×(1 set x 8–12 reps of 8 exercises).wk^-1^ supervised	AT-M: 89 (74–98%); AT-F: 85 (85–98%); RT-M: 95 (81–100%); RT-F: 94 (85–98%)
Janssen 2002 [56]	F (38)	37	33	4 mo.	100%	Metabolic risk factors	1,000 kcal.day^-1^ deficit; <30% kcal from fat.	AT: 5×34 min.wk^-1^ supervised; RT: 3×(1 set x 8–12 reps of 8 exercises).wk^-1^ supervised	AT: 92 (85–98%); RT: 94 (79–100%)
Rice 1999 [58]	M (29)	44	33	4 mo.	88%	Glucose tolerance, insulin action	1,000 kcal.day^-1^ deficit; <30% kcal from fat.	AT: 5×37 min.wk^-1^ supervised; RT: 3×(1 set x 8–12 reps of 8 exercises).wk^-1^ supervised	AT: 92 (74–99%); RT: 96 (85–100%)
Foster-Schubert 2012 [55]	F (204)	58	30.9	12 mo.	91%	Body mass	1,200–2,000 kcal.day^-1^; <30% kcal from fat.	AT: 5×45 min.wk-1; ≥ 3 sessions supervised.	85%

* Macronutrient composition (%carbohydrate/%protein/%fat). bw, body weight; RT, resistance training; AT, aerobic training; F, female; M, male; ME, moderate intensity exercise group; VE, vigorous intensity exercise group; ER, energy restriction; HP, high protein diet; STD, standard diet; Energy restriction relate to energy deficit associated with dietary modification and do not include additional energy expenditure from the addition of exercise.

### Characteristics of included studies

The studies that were included are described in Table 2. The mean age of participants across all studies ranged from 37–75years with mean body mass index (BMI) ranging from 31 kg.m^2^to 37 kg.m^2^. Intervention duration ranged from three to twelve months. There were no exercise training studies that used restrictive bariatric surgery as a form of energy restriction and therefore all studies employed dietary energy restriction. Energy deficits ranged between 400 kcal.d^-1^ and 1,000 kcal.d^-1^ across the 14 studies. The exercise training interventions included progressive resistance training (RT), aerobic training (AT) or a combination of the two modes. AT was predominately performed at moderate to vigorous intensities (65–85% maximum or peak heart rate) for between 90 and 225 minutes per week distributed over three to five days. RT protocols typically utilised two to three sets of 8–12 repetitions for eight to nine different exercises on alternate days of the week at approximately 65% of one repetition maximum (1-RM), progressing to approximately 85% 1-RM. Three studies [56], [57], [58] assessed strength in the exercise training group only and therefore these results will be omitted from this review.

One study [59] reported on results at six months following weight loss and again six months later after weight maintenance. Only the data from the energy restriction period (initial six months) of the study was included for the purpose of this review. Ten studies [52], [55], [59], [60], [61], [62], [63], [64], [65], [66] included older adults and postmenopausal women aged 50-75, while four studies included younger participants aged 37 to 44 years [56], [57], [58], [67]. All studies excluded the use of hormone replacement therapy and two of the 12 studies allowed the continued use of anti-diabetic and anti-hypertensive medication[52], [64]. Seven studies specifically excluded those with diabetes [55], [60], [61], [62], [63], [65], [67], while four allowed inclusion of participants with type 2 diabetes[52], [59], [64], [66]. One study did not prevent or exclude the use of statin therapy during the intervention [64].

### Adverse Events

Two studies reported on adverse events [59], [64] while 12 studies did not report on adverse events [52], [55], [56], [57], [58], [60], [61], [62], [63], [65], [66], [67]. One study found no adverse events related to participation [64] but reported the drop out of two volunteers due to adverse reactions to medications. The other study [59] to report on adverse events that were probably related or definitely related to study participation include: seven complaints of back or knee pain in the exercise training groups; one ankle fracture; one tendon tear and tendonitis; one hematoma; and one paroxysm of atrial fibrillation during exercise [59]. Light headedness or dizziness were also reported for participants in each arm of this study [59]. In addition, the investigators reported other adverse outcomes that they suggested were unrelated to study participation including back and hernia surgery, total hip arthroplasty, three reports of falls and a paroxysm of atrial fibrillation at home.

### Risk of Bias within studies

Assessment of study quality and risk of bias is shown in Table 3 based on generic quality features. Blinding of the outcome assessor was not possible in the studies included or not noted. All studies used objective standardised outcome measures and assessed accordingly. There were no analyses of the risk of bias across studies due to the lack of included publications in this review.

**Table 3:** Appraisal of the quality of studies included.

Reference	Method of treatment assignment	Control of selection bias after treatment assignment	Blinding	Outcome assessment (if blinding was not possible)
Villareal 2011 [59]	b	b	c	a
Nicklas 2009 [64]	a	b	c	a
Toledo 2008 [67]	b	c	c	a
Straznicky 2010 [65]	a	c	c	a
Amati 2008 [60]	c	c	c	a
Messier 2010 [63]	b	d	c	a
Frimel 2008 [62]	b	c	c	a
Daly 2005 [52]	b	c	c	a
Wycherley 2010 [66]	b	c	c	a
Bouchard 2009 [61]	b	c	c	a
Janssen 1999 [57]	b	c	c	a
Janssen 2002 [56]	b	c	c	a
Rice 1999 [58]	b	c	c	a
Foster-Schubert 2012 [55]	a	a	c	a

### Cardio-respiratory fitness

Ten of the 14 studies (Table 4) assessed VO_2peak_ using either a treadmill [55], [56], [57], [58], [59], [64], [67] or cycle protocol [60], [63], [65]. VO_2peak_ was variously expressed in absolute terms, ml.min^-1^ [55], [56], [57], [58], [63], [64], and relative to total body mass, ml.min^-1^.kg BM^-1^ [59], [64] and lean body mass, ml.min^-1^.kg LM^-1^ [60], [65], [67]. Two studies showed change in cardiovascular fitness for the aerobic training groups but not their respective diet only and resistance training groups [56], [58]. Four of the seven studies showed the energy restriction plus exercise training (aerobic only or combined training) groups to significantly improve cardiovascular fitness compared to energy restriction alone [55], [59], [60], [64], [65], whilst two did not show a difference between groups when using aerobic only or resistance training [63], [67].

**Table 4:** Changes in absolute and relative cardiovascular fitness following energy restriction and energy restriction with exercise training.

Reference		Δ Absolute VO_2peak_ (SD) ml.min^-1^	Δ Absolute VO_2peak_ Relative to baseline (%)	Δ Relative VO_2peak_ (SD) ml.min^-1^.kg BM^-1^	Δ VO_2peak_.kg BM relative to baseline (%)	Δ Relative VO_2peak_ (SD) ml.min^-1^.kg LM^-1^	Δ VO_2peak_.kg LM relative to baseline	p-value between group difference for reported outcome measure
Villareal 2011 [59]	ER:	−26*	−1.4*	1.4 (1.7)^#^	8.0*	1.4*	4.6*	(2) p = 0.01
	ER+E:	123*	7.2*	2.8 (2.3)^#^	16.2*	3.1*	10.4*	
Nicklas 2009 [64]	ER:	−71 (255)	−3.8*	2.0 (2.6)^§^	9.6(11.2) ^§^	1.5*	4.2*	ER+VE greater than others; (2) p = 0.03
	ER+ME:	−8 (213)	−0.4*	2.5 (2.6)^§^	12.7(12.7) ^§^	2.3*	6.4*	
	ER+VE:	68 (252)	3.8*	4.1 (3.7)^§^	24.2(27.6) ^§^	3.8*	11.0*	
Toledo 2008 [67]	ER:	−58*	−2.5*	0.42*	9.8*	0.7 (NR)	1.6(4.0)	(3) Not significant
	ER+E:	184*	7.4*	2.85*	17.9*	4.8 (NR)^§^	10.7(3.9)	
Straznicky 2010 [65]	ER:	−56*	−3.9*	−0.18*	4.0*	−0.3 (0.22)	−1.1*	(3) p<0.05
	ER+E:	240*	15.5*	3.15*	27.0*	5.1 (0.25)^#^	17.5*	
Amati 2008 [60]	ER:	−52*	−8.7*	0.08*	20.4*	0.1 (NR)	0.4*	(3) p<0.05
	ER+E:	46*	1.3*	1.06*	31.5*	1.4 (NR)^§^	4.3*	
Messier 2010 [63]	ER:	−100 (NR)^§^	−6.7*	−0.09*	−0.5*	−1.51*	−4.6*	(1) Not significant
	ER+E:	−100 (NR)^§^	−6.7*	0.04*	0.3*	−1.9*	−5.6*	
Janssen 1999 [57]	ERm:	140 (150)	NR	NR	NR	NR	NR	(1) ER+AM >ER+AF p<0.01
	ERf:	−200 (100)	NR	NR	NR	NR	NR	
	ER+AM:	470 (131)^¥^	17.0 (5) ^¥^	NR	NR	NR	NR	
	ER+AF:	200 (50)^¥^	9 (3) ^¥^	NR	NR	NR	NR	
	ER+RM:	180 (110)	NR	NR	NR	NR	NR	
	ER+RF:	110 (150)	NR	NR	NR	NR	NR	
Rice 1999 [58]	ER:	NR	NR	NR	NR	NR	NR	NR
	ER+AT:	NR	16.1 (14.7)^§^	NR	NR	NR	NR	
	ER+RT:	NR	NR	NR	NR	NR	NR	
Janssen 2002 [56]	ER:	NR	NR	NR	NR	NR	NR	NR
	ER+AT:	NR	9 (9)^§^	NR	NR	NR	NR	
	ER+RT:	NR	NR	NR	NR	NR	NR	
Foster-Schubert 2012 [55]	ER:		−1.0 (NR)	1.6*	1.9*	0.02*	0.04*	(1) p = 0.0001
	ER+AT:	120 (340) ^§^	6.5 (NR) ^§^	4.4*	5.3*	3.3*	6.82*	

ER, energy restriction; ER+E, energy restriction and exercise; ER+ME, energy restriction and moderate intensity exercise; ER+VE, energy restriction and vigorous exercise; ER+AT, energy restriction and aerobic training; ER+RT, energy restriction and resistance training; ER+AM male energy restriction and aerobic exercise training; ER+AF female energy restriction and aerobic exercise training; ERm, male energy restriction; ERf, female energy restriction; ER+RM, male energy restriction and resistance training; ER+RF, female energy restriction and resistance training. *Data estimated by calculating from group means (see Methods for more on this). Reported outcome measures: (1) Absolute VO_2_; (2) VO_2_ relative to BM; (3) VO_2_ relative to LM. NR not reported or not able to be calculated. ^§^ denotes within group change (p<0.05). ^¥^ denotes within group change (p<0.01). ^∫^ denotes within group change (p = 0.02). ^#^ denotes significant within group change (p<0.001).

All nine aerobically trained groups showed a significant improvement in either reported absolute or relative VO_2peak_. The only resistance training group showed a significant decrease in VO_2peak_ from baseline but was not significantly different to their respective energy restriction only group [63]. Two studies showed a significant VO_2peak_ improvement relative to total body mass in both energy restriction alone and energy restriction plus exercise training groups when training consisted of either combined aerobic and resistance training [59] or aerobic training alone [64]. The exercise training groups in these studies showed a significantly greater improvement than dieting alone [56], [57], [58].

### Muscle strength

Three studies assessed both upper and lower extremity strength [52], [59], [62], while three others assessed either upper [66] or lower [61], [63] extremity strength alone. Five of these studies [52], [59], [62], [63], [66] used the one repetition maximum (1-RM) strength test while one study used isometric leg extension with knee joint angle at 90 degrees [61]. All exercise training interventions demonstrated improved strength both within groups and between groups (energy restriction plus exercise training versus respective energy restriction alone), with the exception of the study of isometric strength [61] where there was a slight decrease in strength performance. All energy restriction alone groups showed negligible change or decreases in strength following weight loss. [59], [63], [66] Resistance training or combined resistance and aerobic training improved isotonic strength compared with their respective diet only groups [52], [59], [62], [63], [66]. In the only study that assessed isometric strength, there was a decrease in strength of 15% in energy restriction plus exercise training and 25% in energy restriction alone with no between group difference [61].

### Capacity to perform activities of daily living

There is little data on the effects of exercise training added to energy restriction in terms of capacity to perform activities of daily living. One study[59] evaluated participants' physical function with the use of the modified Physical Performance Test (a selection of seven standardised tasks including walking 50ft, putting on and removing a coat, picking up a coin, standing up from a chair, lifting a book, climbing a flight of stairs, and a progressive Romberg test) and two additional tests of climbing up and down four flights of stairs and a 360 degree turn. The physical performance test score increased by 21% and 12% in the energy restriction plus aerobic and resistance exercise training group and energy restriction group respectively. This study showed changes in single-leg balance at six months improved by 6.3 sec in energy restriction plus exercise training and by 0.8 sec in energy restriction alone. Gait speed increased by 5.5 m.min^-1^ and 1.7 m.min^-1^ in energy restriction plus exercise training and energy restriction alone. Improved obstacle course time was reported in both energy restriction plus exercise training (−1.1 sec) and energy restriction alone (−0.7 sec). A second study [61] assessed physical function using a selection of similar tests but did not show a significant difference between groups in performance following either energy restriction alone or energy restriction plus resistance exercise training interventions.

### Body mass loss and body composition

The 14 studies included in this review reported on 16 energy restriction only groups and 21 energy restriction plus exercise training groups (Table 5). The mean body mass loss across studies of three to four months in duration ranged from 4 ±NR (not reported) kg to 12.1±3.4 kg and 3.6±NR kg to 13.6±4.1 kg in the energy restriction and energy restriction plus exercise training groups respectively [56], [57], [58], [60], [61], [65], [66]. In the longer five and six month studies the mean body mass loss ranged from 3±1.8 kg to 10.7±4.5 kg and 2.4±3.0 kg and 9.7±4.0 kg in energy restriction and energy restriction plus exercise training groups respectively [52], [59], [62], [63], [67]. For the only 12 month long study there was a mean body mass loss of 7.1±NR kg and 8.9±NR kg in the energy restriction and energy restriction plus exercise training groups [55]. There were no significant between group difference in body mass loss within the studies, with the exception of two [55], [66] (Table 5). One of these [55] studies reported increased physical activity and fat mass loss in the energy restriction plus aerobic exercise training group compared to the energy restriction only group. There were no differences in energy intake between groups. The remaining study [66] reported a greater fat mass loss in the energy restriction plus exercise training groups compared with the energy restriction only groups with no difference in energy intake. This study did not report on the exercise participation for the energy restriction only groups. The exercise groups completed 93% of the planned 45 minute resistance training sessions three days per week. Fat mass loss between the energy restriction only and energy restriction plus exercise training groups was not different in those studies that showed total body mass loss between groups to be similar. Five studies showed that lean mass or skeletal muscle mass was lost more in the energy restriction only groups compared to the exercise training groups but this was not sufficient to produce a significant difference in total mass loss between the groups [52], [58], [59], [60], [62]. The studies where there was no difference in total mass loss between the energy restriction only and energy restriction plus exercise training did not compare energy deficit differences [52], [59], [60], [61], [62], [63], [64], [65], [67] or reported similar energy deficits between groups [56], [57], [58].

**Table 5:** Changes in weight, body composition, fitness and function for all included studies.

Reference		Δ Body weight Kg (SD)	Δ Body weight %	Δ Lean mass Kg (SD)	Δ Lean mass %	Δ Fat mass Kg (SD)	Δ Fat mass %	Reported VO_2peak_	Strength
Foster-Schubert 2012 [55]	ER:	−7.2 (NR)^#^a	−8.5	−0.8 (NR)	−1.9^#^	−6.1 (NR)	−15.6^#^a	↔ a	
	ER+AT	−8.9 (NR)^#^b	−10.8	−0.4 (NR)	−1.1	−8.2 (NR)	−20.8^#^b	↑b	
Villareal 2011 [59]	ER:	−9.0 (5.4)^#^	−8.6	−3.5 (2.7)^#^a	−5.7	−6.0 (3.8)^#^	−14.0	↓ a	↔ a
	ER+E:	−7.7 (4.2)^#^	−7.7	−1.7 (1.7)^#^b	−3.0	−5.6 (3.2)^#^	−13.4	↑b	↑b
Nicklas 2009 [64]	ER:	−11.8 (4.1)^#^	−12.9	−4.1 (1.9)^#^	−7.7	−7.4 (2.8)^#^	−18.6	↓a	
	ER+ME:	−12.2 (4.5)^#^	−13.5	−3.4 (2.0)^#^	−6.5	−8.2 (3.2)^#^	−20.9	↔	
	ER+VE:	−12.3 (4.9)^#^	−13.9	−3.3 (1.7)^#^	−6.4	−8.5 (3.8)^#^	−22.0	↑b	
Toledo 2008 [67]	ER:	−10.6 (NR)^#^	−11.2	−2.1 (NR)^#^	−4.0	−7.5 (NR)^#^	−19.0	↔	
	ER+E:	−8.5 (NR)^#^	−9.0	−1.3 (NR)	−2.5	−7.1 (NR)^#^	−17.7	↑	
Straznicky 2010 [65]	ER:	−7.1 (2.7)^#^	−7.5	−1.5 (2.2)^#^	−2.8	−5.2 (3.1)^#^	−14.3	↓a	
	ER+E:	−8.4 (4.5)^#^	−9.0	−0.9 (1.8)^#^	−1.7	−6.9 (4.0)^#^	−19.5	↑ b	
Amati 2008 [60]	ER:	−7.3 (NR)^#^	−9.2	−2.0 (NR)^#^a	−4.3	−6.3 (NR)^#^	−16.4	↓a	
	ER+E:	−8.5 (NR)^#^	−8.5	−0.8 (NR)b	−1.5	−6.8 (NR)^#^	−18.7	↑b	
Messier 2010 [63]	ER:	−5.2 (NR)^#^	−6.2	−1.0 (NR)^#^	−2.2	−3.9 (NR)^#^	−10.4	↓	↔ a
	ER+E:	−5.8 (NR)^#^	−6.9	−0.5 (NR)^#^	−1.1	−5.3 (NR)^#^	−13.4	↓	↑b
Frimel 2008 [62]	ER:	−10.7 (4.5)^#^	−10.4	−3.5 (2.1)^#^a	−5.9	−6.8 (3.7)^#^	−16.1		↔ a
	ER+E:	−9.7 (4.0)^#^	−10.0	−1.8 (1.5)b	−3.1	−7.7 (2.9)^#^	−19.5		↑b
Daly 2005 [52]	ER:	−3.0 (1.8)^#^	−3.5	−0.4 (1.0)a	−0.03	−2.0 (2.3)^#^	−6.5		↔ a
	ER+E	−2.4 (3.0)^#^	−2.8	0.5 (1.1)b	1.0	−2.4 (2.9)^#^	−7.2		↑b
Wycherley 2010 [66]	ERs:	−8.6 (4.6) ^§#^a	−8.9	−2.2 (1.9)	−3.8	−6.5 (3.7) ^§#^a	−16.6		↔ a
	ERhp:	−9.0 (4.8)	−10.0	−1.9 (1.5)	−3.7	−7.1 (4.0)	−20.1		↓a
	ERs+E:	−10.5 (5.1) ^§#^b	−8.8	−2.4 (2.5)	−3.0	−8.1 (3.8) ^§#^b	−17.8		↑b
	ERhp+E:	−13.8 (6.0)	−12.8	−2.4 (3.1)	−3.7	−11.4 (3.9)	−26.6		↑b
Bouchard 2009 [61]	ER:	−4.0 (NR)^#^	−5.1	−0.7 (NR)^#^	−1.8	−3.2 (NR)^#^	−8.9		↓
	ER+E:	−3.6 (NR)^#^	−4.6	−0.3 (NR)	−0.7	−3.2 (NR)^#^	−9.5		↓
Janssen 1999 [57]	ERm:	−11.7 (3.5)^#^	−11.9	−2.9 (1.9)^#^	−4.4	−7.5 (2.8)^#^	−24.2	↔	
	ERf:	−10.7 (3.8)^#^	−11.5	−1.5 (1.3)^#^	−3.2	−8.2 (3.5)^#^	−19.2	↔	
	ER+AM:	−11.4 (3.8)^#^	−11.2	−1.6 (1.6)	−2.5	−9.4 (3.8)^#^	−27.9	↑	
	ER+AF:	−11.5 (3.2)^#^	−11.7	−0.5 (1.9)	−1.0	−10.5 (3.8)^#^	−22.6	↑	
	ER+RM:	−12.7 (3.8)^#^	−11.6	−1.8 (2.5)	−2.7	−10.3 (3.8)^#^	−27.5	↔	
	ER+RF:	−10.0 (2.8)^#^	−11.5	−0.2 (1.9)	−0.4	−8.7 (2.2)^#^	−22.4	↔	
Janssen 2002 [56]	ER:	−10.0 (3.9)^#^	−11.0	SM-1.1 (0.8)^#^	−4.8	−7.8 (3.1)^#^	−18.9	↔	
	ER+AT:	−11.1 (4.4)^#^	−11.1	SM-0.6 (1.1)	−2.5	−9.9 (4.6)^#^	−20.9	↑	
	ER+RT:	−10.0 (3.0)^#^	−11.6	SM-0.4 (1.1)	−1.9	−8.6 (2.4)^#^	−22.8	↔	
Rice 1999 [58]	ER:	−12.1 (3.4)^#^	−12.2	SM -2.5 (1.0L)^#^a	−7.2	−8.5 (2.9L)^#^	−24.9	↔	
	ER+AT:	−11.5 (3.9)^#^	−11.4	SM +0.3 (1.0L)^#^b	0.9	−9.7 (4.6L)^#^	−28.1	↑	
	ER+RT:	−13.6 (4.1)^#^	−12.4	SM +0.2 (2.2L)^#^b	0.6	−10.8 (3.5L)^#^	−27.3	↔	

ER, energy restriction; ER+E, energy restriction and exercise; ER+ME, energy restriction and moderate intensity exercise; ER+VE, energy restriction and vigorous exercise; ERs, energy restriction standard diet; ERhp, energy restriction high protein diet; ER+AT, energy restriction and aerobic training; ER+RT, energy restriction and resistance training; ER+AM male energy restriction and aerobic exercise training; ER+AF female energy restriction and aerobic exercise training; ERm, male energy restriction; ERf, female energy restriction; ER+RM, male energy restriction and resistance training; ER+RF, female energy restriction and resistance training. ^#^ significant within group changes with intervention. Values with different letters (^a,^ ^b^) significant between group changes with intervention. ^§^significant difference between combined diet only interventions and combined exercise and diet interventions.NR not reported or not able to be calculated. ↑significant improvement within group; ↓significant decrease within group; ↔no change within group. SM, skeletal muscle mass. Reported VO_2peak_ shows relative VO_2peak_. Absolute VO_2peak_ is shown if the study did not report relative VO_2peak_.

In the energy restriction only groups, lean mass or skeletal muscle mass was reduced in 15 of the 16 diet only groups [55], [56], [57], [58], [59], [60], [61], [62], [63], [64], [65], [66], [67], and nine of the 21 combined energy restriction plus exercise training groups (Table 5). Lean mass did not change in the energy only group in one study [52] where energy restriction achieved a modest 3.5% total mass loss after six months. Lean mass loss in energy restriction groups ranged from 0.4±1.0 kg to 4.1±1.9 kg while lean mass loss in energy restriction plus exercise training ranged from a gain of 0.5±1.1 kg to a loss of 3.4±2.0 kg. Skeletal muscle mass was also shown to increase in one study [58] using MRI after four months with either aerobic or resistance training. Six studies reported that the energy restriction plus exercise training groups lost significantly less lean mass or skeletal muscle mass than the corresponding energy restriction only groups [52], [58], [59], [60], [62].

Predicted means of the reported percentage changes from baseline, in treatment means for body mass, lean mass, and fat mass are presented in Table 6. The predicted means constructed from the estimated effects in the linear mixed model analysis were unadjusted against covariates such as energy imbalance or change in total body mass. The summaries in Table 6 exclude the two studies [42], [44] that reported skeletal mass and some assumptions were made about the data from two other studies [60], [64]. For Nicklas et al. [64] we assumed that the baseline (pre) means were not significantly changed by the drop-out of 17 (15%) of the 112 participants. For Amati et al. [60] we interpreted the footnote to their Table 4 as “means of changes were divided by the overall baseline mean” rather than each participant's change was divided by each participant's baseline value and these ratios were subsequently averaged - the former interpretation is consistent with the analysis (a repeated measures Anova) described in the Methods section of their paper. Predicted means of the percentage changes from baseline for each treatment for body mass were not significantly different across treatments (Table 6).There was significant variation in the predicted treatment means of the percentage changes in lean mass. The loss in lean mass was greater in the energy restriction alone treatment (−3.61±0.61%) compared to the energy restriction with the addition of aerobic exercise training (−2.18±0.66%), resistance training (−2.49±0.67%), and combined aerobic and resistance exercise training (−1.15±0.9%). There was significant variation in the predicted treatment means of the percentage changes in fat mass. The LSD tests revealed that the fat mass loss with energy restriction alone (−14.77±1.48%) was less than the energy restriction plus aerobic exercise training or resistance training interventions, but not in the combined aerobic and resistance exercise training groups. The calculated proportion of lean mass loss to total mass loss ranged from 11.3% to 38.9% and 2.0% to 27.9% in energy restriction alone and the collective energy restriction plus exercise training groups respectively.

**Table 6:** Predicted means, over studies, of percentage changes relative to their baseline values in lean mass (LM), fat mass (FM) and body mass (BM) means.

Variable	ER (n = 14)	ER+A (n = 8)	ER+R (n = 7)	ER+A+R (n = 2)	F-test p-value
LM	−3.61±0.61 a	−2.18±0.66 b	−2.49±0.67 b	−1.15±0.90 b	<0.001
FM	−14.77±1.48 b	−18.28±1.67 a	−17.69±1.72 a	−16.20±2.58 a,b	0.025
BM	−8.59±0.85	−9.03±0.91	−9.13±0.93	−8.05±1.25	0.622

ER =  Energy Restriction, ER+A =  Energy Restriction + Aerobic Training, ER+R =  Energy Restriction + Resistance Training, and, ER+A+R =  Energy Restriction + a combination of Aerobic and Resistance Training. Plus-minus values are predicted means and their standard errors from the linear mixed model analysis. Within a row, corresponding to a variable, predicted means with lower-case letters in common are not significantly different on the basis of LSDs. n is the number of groups exposed to a certain treatment; excludes 2 studies that reported skeletal mass.

## Discussion

This review documents evidence that exercise training provides important benefits for obese adults undergoing energy restriction in regards to cardiovascular fitness, muscle strength, and body composition. The 14 studies documented a total mass loss range of 3.0 to 12.1 kg for energy restriction alone and 2.4 to13.6 kg for energy restriction combined with exercise training. In this collection of studies there is a smaller decrease in relative lean mass (approximately 1 to 2%) when either aerobic, resistance or combined exercise training is used in addition to energy restriction. Greater reductions in relative fat mass (approximately 3%) is achieved when either aerobic or resistance exercise training alone, but not combined exercise training is added to energy restriction.

The capacity to perform ADLs is influenced by many factors including BMI, muscle strength, VO_2peak_, and age, and may also be linked to the number of hours of TV watching [68]. Obesity results in reduced aerobic capacity, muscle strength, functional capacity and lower fatigue resistance when compared to non-obese individuals [10], [69], [70]. This review provides evidence that weight loss in the absence of regular exercise training has no or limited benefits for muscle strength, whilst exercise training during energy restriction improves strength. Exercise training during energy restriction appears to improve cardiovascular fitness to a greater extent than energy restriction alone. These changes in fitness will almost certainly improve functional capacity which is impaired in obesity [59], [71], [72]. The physical performance test was evaluated by two studies in this review [59], [61]. An accumulated score of all measured items showed that fat mass loss may be related to improved functional capacity; however the addition of regular exercise training further facilitates improvement and may be explained by improved muscle strength, VO_2peak_, and the protection of lean mass in this group of frail older adults. The study by Bouchard and colleagues [61] showed greater improvements in one-leg squat, 6-minute walk, and one-leg stand in the resistance training groups compared to energy restriction alone but did not show greater improvements on the global physical capacity scale when comparing diet alone to diet combined with exercise training. Exercise training alone in this study did show greater overall physical performance tests compared to the diet only and the combined diet and exercise training group. Limitations of that study may include a small sample size, short intervention period (3 months) and limited weight loss. Despite a lack of convincing evidence from Bouchard and colleagues [61], it appears that physical capacity is further enhanced when exercise training is combined with weight loss. This is particularly evident in those tasks that require muscle strength and power.

Previous studies have shown that energy restriction combined with exercise training sufficient to evoke weight loss can promote improved functional capacities, work capacity and cognitive performance to a greater extent than energy restriction loss alone [21], [73], [74], [75], [76]. The risk of musculoskeletal injury may be further reduced and functional capacity enhanced if the training stimulus is sufficient to stimulate improvement in strength and aerobic capacity [8], [17], [29]. Five studies included in this review showed that resistance exercise training included either in isolation or when combined with aerobic exercise training during energy restriction stimulated strength improvements of between 20–30% in 4–6 months, and could lead to improved occupational performance [75], [77] and reduce occupational musculoskeletal injury [78].

VO_2peak_ is related to the capacity to perform activities of daily living in obese individuals [11] and is a predictor of occupational injury risk [79]. Aerobic exercise training and combined aerobic and resistance training improve cardiovascular fitness compared to energy restriction alone. Energy restriction with and without resistance training in one study [63] showed a decrease in absolute VO_2peak_ but did not report changes in relative VO2peak. Combining aerobic and resistance training improved cardiovascular fitness in energy restriction with exercise training compared to energy restriction alone [59] but not to the extent of improvements seen in those that used aerobic training alone [60], [64], [65], [67]. The larger differences in VO_2peak_ were found when VO_2peak_ was measured relative to total mass or lean mass, with only small differences for absolute measures of VO_2peak_. This is functionally important because power to weight ratio predicts the performance of weight bearing exercise better than absolute power.

Across all studies there is a smaller decrease in lean mass and greater decrease in fat mass when either aerobic or resistance training is added to energy restriction. Only two of the 31 groups in the review of 14 studies used combined aerobic and resistance training and this explains the higher estimated standard errors for predictions of changes in body mass and composition for this treatment and, with regard to fat mass, this may also explain the non-significant comparison with the ER treatment. Two studies were excluded from analysis as they used measures of skeletal muscle mass rather than global lean mass. One study showed a within group and between group increase in skeletal muscle mass [58]. The remaining study showed that skeletal muscle mass remained unchanged after the exercise training interventions [56].

Excessive loss of lean mass may be related to premature mortality [80]. Webster et al. [81] suggests that lean mass loss should not comprise more than 22% of total weight loss. Resistance or aerobic exercise training during energy restriction can meet such a target during weight loss, while it appears that energy restriction alone cannot and is therefore an important reason for including exercise training in weight loss interventions.

Obesity, lack of regular exercise training, and low cardiovascular fitness and muscle strength are modifiable factors that relate to declining physical capacity. The studies included in this review suggest that the addition of regular exercise training during energy restriction facilitates improvements in physical performance to a greater extent than energy restriction alone. More favourable changes to body composition, strength and VO_2peak_ observed in the exercise trained groups led to improved functional capacities.

This review was limited by the heterogeneity of the frequency, intensity, duration and mode of the exercise training approaches employed across studies. The exercise variables are used to modify or induce physiological adaptations including aerobic fitness, muscular strength, endurance or hypertrophy The degree of energy restriction and macro-nutrient composition of the diet varied across studies which is likely to influence the rate of lean mass and body mass loss [27], [82]. There was heterogeneity of age of participants (mean 56 yr; range 37–75 yr), medication usage, and co-morbidities which limits the generalisability of the current findings. Additional studies are warranted that focus on younger obese populations with longer follow up

A further limitation is that the linear mixed model analyses of changes in body mass and composition were unweighted as the information required to calculate appropriate weights was not available. A weighted analysis would have used the estimated variances of each of the percentage changes and these estimates, based on the delta method, would have required the variances and covariances of the pre and post means of each group. Nevertheless, the unweighted analyses enabled us to estimate treatment effects from the combined reports of the studies.

Despite the limitations of this review, the addition of exercise training during energy restriction positively influence body composition, cardiovascular and muscular fitness. These combined benefits of exercise training with energy restriction may influence injury risk, postpone onset and severity of physical disability and may predict functional capacity later in life. The reduction of disability risk in the obese during aging may best be managed by the modification of diet with the inclusion of tailored exercise training that promotes improvements in physical conditioning in addition to the reduction of fat mass and protection of lean mass. Although weight loss itself is an important outcome, obese individuals and their health carers should be encouraged to look beyond weight loss as the only outcome goal.

In conclusion, this systematic review demonstrates that exercise training confers benefits in absolute and relative cardiovascular fitness, muscle strength; and positive body composition outcomes in middle-aged and older adults who are attempting to lose weight by energy restriction. Exercise training promotes greater fat mass loss and assists the preservation of lean mass, compared to energy restriction alone during weight loss interventions. Further, energy restriction without exercise training has a neutral or negative influence on cardiorespiratory fitness, muscular strength, and generates greater loss of lean mass. Clinicians should recommend exercise training as part of a lifestyle modification for obese individuals with a focus on improving fitness, function and lean mass rather than merely creating greater energy deficit for weight loss.

## Supporting Information

Checklist S1 PRISMA Checklist.
(DOC)

## References

[1] Haslam DW, James WP (2005) Obesity. Lancet 366: 1197–1209. 16198769 10.1016/S0140-6736(05)67483-1

[2] Dixon JB (2010) The effect of obesity on health outcomes. Molecular and Cellular Endocrinology 316: 104–108. 19628019 10.1016/j.mce.2009.07.008

[3] Peppard PE, Young T, Palta M, Dempsey J, Skatrud J (2000) Longitudinal study of moderate weight change and sleep-disordered breathing. JAMA 284: 3015–3021. 11122588 10.1001/jama.284.23.3015

[4] Formiguera X, Cantón A (2004) Obesity: epidemiology and clinical aspects. Best Practice & Research Clinical Gastroenterology 18: 1125–1146. 15561643 10.1016/j.bpg.2004.06.030

[5] Anandacoomarasamy A, Fransen M, March L (2009) Obesity and the musculoskeletal system. Curr Opin Rheumatol 21: 71–77. 19093327 10.1097/bor.0b013e32831bc0d7

[6] Leone N, Courbon D, Berr C, Barberger-Gateau P, Tzourio C, et al.. (2011) Abdominal Obesity and Late-Onset Asthma: Cross-Sectional and Longitudinal Results: The 3C Study. Obesity (Silver Spring). 10.1038/oby.2011.308 22016095

[7] Bouchard DR, Langlois MF, Brochu M, Dionne IJ, Baillargeon JP (2011) Metabolically healthy obese women and functional capacity. Metab Syndr Relat Disord 9: 225–229. 21361821 10.1089/met.2010.0101

[8] Hills AP, Hennig EM, Byrne NM, Steele JR (2002) The biomechanics of adiposity—structural and functional limitations of obesity and implications for movement. Obes Rev 3: 35–43. 12119658 10.1046/j.1467-789x.2002.00054.x

[9] Jebb SA, Moore MS (1999) Contribution of a sedentary lifestyle and inactivity to the etiology of overweight and obesity: current evidence and research issues. Med Sci Sports Exerc 31: S534–541. 10593524 10.1097/00005768-199911001-00008

[10] Maffiuletti NA, Jubeau M, Munzinger U, Bizzini M, Agosti F, et al (2007) Differences in quadriceps muscle strength and fatigue between lean and obese subjects. Eur J Appl Physiol 101: 51–59. 17476522 10.1007/s00421-007-0471-2

[11] He XZ, Baker DW (2004) Body mass index, physical activity, and the risk of decline in overall health and physical functioning in late middle age. Am J Public Health 94: 1567–1573. 15333316 10.2105/ajph.94.9.1567 PMC1448495

[12] Stenholm S, Strandberg TE, Pitkala K, Sainio P, Heliovaara M, et al.. (2013) Midlife Obesity and Risk of Frailty in Old Age During a 22-Year Follow-up in Men and Women: The Mini-Finland Follow-up Survey. J Gerontol A Biol Sci Med Sci. 10.1093/gerona/glt052 23640762

[13] Stenholm S, Alley D, Bandinelli S, Griswold ME, Koskinen S, et al (2009) The effect of obesity combined with low muscle strength on decline in mobility in older persons: results from the InCHIANTI study. Int J Obes (Lond) 33: 635–644. 19381155 10.1038/ijo.2009.62 PMC2697265

[14] Capodaglio P, Castelnuovo G, Brunani A, Vismara L, Villa V, et al (2010) Functional limitations and occupational issues in obesity: a review. Int J Occup Saf Ergon 16: 507–523. 21144269 10.1080/10803548.2010.11076863

[15] Vieira ER, Kumar S, Narayan Y (2008) Smoking, no-exercise, overweight and low back disorder in welders and nurses. International Journal of Industrial Ergonomics 38: 143–149.

[16] Monteiro MS, Alexandre NMC, Milani D, Fujimura F (2011) Work capacity evaluation among nursing aides. Revista da Escola de Enfermagem da USP 45: 1177–1182. 10.1590/s0080-62342011000500021 22031380

[17] Janssen I, Bacon E, Pickett W (2011) Obesity and its relationship with occupational injury in the canadian workforce. J Obes 2011: 531403. 21773008 10.1155/2011/531403 PMC3136224

[18] Blazer DG, Hybels CF, Fillenbaum GG (2006) Metabolic syndrome predicts mobility decline in a community-based sample of older adults. J Am Geriatr Soc 54: 502–506. 16551320 10.1111/j.1532-5415.2005.00607.x

[19] Dishman RK, Berthoud HR, Booth FW, Cotman CW, Edgerton VR, et al (2006) Neurobiology of exercise. Obesity (Silver Spring) 14: 345–356. 16648603 10.1038/oby.2006.46

[20] Vaynman S, Gomez-Pinilla F (2006) Revenge of the “sit”: how lifestyle impacts neuronal and cognitive health through molecular systems that interface energy metabolism with neuronal plasticity. J Neurosci Res 84: 699–715. 16862541 10.1002/jnr.20979

[21] Coulson JC, McKenna J, Field M (2008) Exercising at work and self-reported work performance. International Journal of Workplace Health Management 1: 176–197.

[22] Garber CE, Blissmer B, Deschenes MR, Franklin BA, Lamonte MJ, et al. (2011) Quantity and Quality of Exercise for Developing and Maintaining Cardiorespiratory, Musculoskeletal, and Neuromotor Fitness in Apparently Healthy Adults: Guidance for Prescribing Exercise. Medicine & Science in Sports & Exercise 43 : 1334–1359 1310.1249/MSS.1330b1013e318213fefb. 10.1249/MSS.0b013e318213fefb 21694556

[23] Weinheimer EM, Sands LP, Campbell WW (2010) A systematic review of the separate and combined effects of energy restriction and exercise on fat-free mass in middle-aged and older adults: implications for sarcopenic obesity. Nutr Rev 68: 375–388. 20591106 10.1111/j.1753-4887.2010.00298.x

[24] Bray GA, Gray DS (1988) Obesity. Part I—Pathogenesis. West J Med 149: 429–441. 3067447 PMC1026489

[25] Kaila B, Raman M (2008) Obesity: a review of pathogenesis and management strategies. Can J Gastroenterol 22: 61–68. 18209783 10.1155/2008/609039 PMC2659122

[26] Guyenet SJ, Schwartz MW (2012) Clinical review: Regulation of food intake, energy balance, and body fat mass: implications for the pathogenesis and treatment of obesity. J Clin Endocrinol Metab 97: 745–755. 22238401 10.1210/jc.2011-2525 PMC3319208

[27] Chaston TB, Dixon JB, O'Brien PE (2007) Changes in fat-free mass during significant weight loss: a systematic review. Int J Obes (Lond) 31: 743–750. 17075583 10.1038/sj.ijo.0803483

[28] Marks BL, Rippe JM (1996) The importance of fat free mass maintenance in weight loss programmes. Sports Med 22: 273–281. 8923645 10.2165/00007256-199622050-00001

[29] Weiss EP, Racette SB, Villareal DT, Fontana L, Steger-May K, et al (2007) Lower extremity muscle size and strength and aerobic capacity decrease with caloric restriction but not with exercise-induced weight loss. J Appl Physiol 102: 634–640. 17095635 10.1152/japplphysiol.00853.2006 PMC4376253

[30] Miller SL, Wolfe RR (2008) The danger of weight loss in the elderly. J Nutr Health Aging 12: 487–491. 18615231 10.1007/BF02982710 PMC12892571

[31] Byrne NM, Weinsier RL, Hunter GR, Desmond R, Patterson MA, et al (2003) Influence of distribution of lean body mass on resting metabolic rate after weight loss and weight regain: comparison of responses in white and black women. The American Journal of Clinical Nutrition 77: 1368–1373. 12791611 10.1093/ajcn/77.6.1368

[32] Beavers KM, Lyles MF, Davis CC, Wang X, Beavers DP, et al (2011) Is lost lean mass from intentional weight loss recovered during weight regain in postmenopausal women? Am J Clin Nutr 94: 767–774. 21795437 10.3945/ajcn.110.004895 PMC3155932

[33] Rantanen T, Guralnik JM, Foley D, Masaki K, Leveille S, et al (1999) Midlife hand grip strength as a predictor of old age disability. JAMA 281: 558–560. 10022113 10.1001/jama.281.6.558

[34] Rantanen T, Guralnik JM, Sakari-Rantala R, Leveille S, Simonsick EM, et al (1999) Disability, physical activity, and muscle strength in older women: the Women's Health and Aging Study. Arch Phys Med Rehabil 80: 130–135. 10025485 10.1016/s0003-9993(99)90109-0

[35] Vargas CB, Picolli F, Dani C, Padoin AV, Mottin CC (2013) Functioning of Obese Individuals in Pre- and Postoperative Periods of Bariatric Surgery. Obes Surg. 10.1007/s11695-013-0924-0 23515976

[36] Chmelo E, Nicklas B, Davis C, Miller GD, Legault C, et al.. (2012) Physical Activity and Physical Function in Older Adults with Knee Osteoarthritis. J Phys Act Health. 10.1123/jpah.10.6.777 PMC4142418 23307503

[37] Manini TM, Newman AB, Fielding R, Blair SN, Perri MG, et al (2010) Effects of exercise on mobility in obese and nonobese older adults. Obesity (Silver Spring) 18: 1168–1175. 19834467 10.1038/oby.2009.317 PMC3114403

[38] Wilson JM, Marin PJ, Rhea MR, Wilson SM, Loenneke JP, et al (2012) Concurrent training: a meta-analysis examining interference of aerobic and resistance exercises. J Strength Cond Res 26: 2293–2307. 22002517 10.1519/JSC.0b013e31823a3e2d

[39] Donnelly JE, Blair SN, Jakicic JM, Manore MM, Rankin JW, et al (2009) American College of Sports Medicine Position Stand. Appropriate physical activity intervention strategies for weight loss and prevention of weight regain for adults. Med Sci Sports Exerc 41: 459–471. 19127177 10.1249/MSS.0b013e3181949333

[40] Ratamess NA, Alvar BA, Evetoch TK, Housh TJ, Kibler BW, et al. (2009) Progression Models in Resistance Training for Healthy Adults. Medicine & Science in Sports & Exercise 41 : 687–708 610.1249/MSS.1240b1013e3181915670.

[41] Das SK (2005) Body composition measurement in severe obesity. Current opinion in clinical nutrition and metabolic care 8: 602–606. 16205459 10.1097/01.mco.0000171122.60665.5f

[42] Woodrow G (2007) Body composition analysis techniques in adult and pediatric patients: how reliable are they? How useful are they clinically? Peritoneal dialysis international: journal of the International Society for Peritoneal Dialysis 27 Suppl 2S245–249. 17556313

[43] Alvarez VP, Dixon JB, Strauss BJ, Laurie CP, Chaston TB, et al (2007) Single frequency bioelectrical impedance is a poor method for determining fat mass in moderately obese women. Obes Surg 17: 211–221. 17476875 10.1007/s11695-007-9032-3

[44] Dempster P, Aitkens S (1995) A new air displacement method for the determination of human body composition. Med Sci Sports Exerc 27: 1692–1697. 8614327

[45] Fields DA, Higgins PB, Radley D (2005) Air-displacement plethysmography: here to stay. Curr Opin Clin Nutr Metab Care 8: 624–629. 16205463 10.1097/01.mco.0000171127.44525.07

[46] Garrow JS, Summerbell CD (1995) Meta-analysis: effect of exercise, with or without dieting, on the body composition of overweight subjects. European Journal of Clinical Nutrition 49: 1–10. 7713045

[47] Going SB, Massett MP, Hall MC, Bare LA, Root PA, et al (1993) Detection of small changes in body composition by dual-energy x-ray absorptiometry. American Journal of Clinical Nutrition 57: 845–850. 8503351 10.1093/ajcn/57.6.845

[48] Ismail I, Keating SE, Baker MK, Johnson NA (2011) A systematic review and meta-analysis of the effect of aerobic vs. resistance exercise training on visceral fat. Obes Rev. 10.1111/j.1467-789X.2011.00931.x 21951360

[49] Glasziou P (2001) Systematic reviews in health care: a practical guide. New York: Cambridge University Press.

[50] Fruhbeck G, Diez Caballero A, Gil MJ (2004) Fundus functionality and ghrelin concentrations after bariatric surgery. N Engl J Med 350: 308–309. 14724316 10.1056/NEJM200401153500323

[51] Beckman LM, Beckman TR, Earthman CP (2010) Changes in gastrointestinal hormones and leptin after Roux-en-Y gastric bypass procedure: a review. J Am Diet Assoc 110: 571–584. 20338283 10.1016/j.jada.2009.12.023 PMC4284064

[52] Daly RM, Dunstan DW, Owen N, Jolley D, Shaw JE, et al (2005) Does high-intensity resistance training maintain bone mass during moderate weight loss in older overweight adults with type 2 diabetes? Osteoporos Int 16: 1703–1712. 15937634 10.1007/s00198-005-1906-4

[53] Payne RW, Harding SA, Murray DA, Soutar DM, Baird DB, et al.. (2011) The Guide to GenStat Release 14, Part 2: Statistics. Hemel Hempstead: VSN International.

[54] Campbell KL, Foster-Schubert KE, Alfano CM, Wang C-C, Wang C-Y, et al (2012) Reduced-calorie dietary weight loss, exercise, and sex hormones in postmenopausal women: randomized controlled trial. Journal Of Clinical Oncology: Official Journal Of The American Society Of Clinical Oncology 30: 2314–2326. 22614972 10.1200/JCO.2011.37.9792 PMC3675691

[55] Foster-Schubert KE, Alfano CM, Duggan CR, Xiao L, Campbell KL, et al (2012) Effect of diet and exercise, alone or combined, on weight and body composition in overweight-to-obese postmenopausal women. Obesity (Silver Spring) 20: 1628–1638. 21494229 10.1038/oby.2011.76 PMC3406229

[56] Janssen I, Fortier A, Hudson R, Ross R (2002) Effects of an Energy-Restrictive Diet With or Without Exercise on Abdominal Fat, Intermuscular Fat, and Metabolic Risk Factors in Obese Women. Diabetes Care 25: 431–438. 11874926 10.2337/diacare.25.3.431

[57] Janssen I, Ross R (1999) Effects of sex on the change in visceral, subcutaneous adipose tissue and skeletal muscle in response to weight loss. Int J Obes Relat Metab Disord 23: 1035–1046. 10557024 10.1038/sj.ijo.0801038

[58] Rice B, Janssen I, Hudson R, Ross R (1999) Effects of aerobic or resistance exercise and/or diet on glucose tolerance and plasma insulin levels in obese men. Diabetes Care 22: 684–691. 10332666 10.2337/diacare.22.5.684

[59] Villareal DT, Chode S, Parimi N, Sinacore DR, Hilton T, et al (2011) Weight loss, exercise, or both and physical function in obese older adults. N Engl J Med 364: 1218–1229. 21449785 10.1056/NEJMoa1008234 PMC3114602

[60] Amati F, Dube JJ, Shay C, Goodpaster BH (2008) Separate and combined effects of exercise training and weight loss on exercise efficiency and substrate oxidation. J Appl Physiol 105: 825–831. 18617627 10.1152/japplphysiol.90384.2008 PMC2536819

[61] Bouchard DR, Soucy L, Senechal M, Dionne IJ, Brochu M (2009) Impact of resistance training with or without caloric restriction on physical capacity in obese older women. Menopause 16: 66–72. 18779759 10.1097/gme.0b013e31817dacf7

[62] Frimel TN, Sinacore DR, Villareal DT (2008) Exercise attenuates the weight-loss-induced reduction in muscle mass in frail obese older adults. Med Sci Sports Exerc 40: 1213–1219. 18580399 10.1249/MSS.0b013e31816a85ce PMC2650077

[63] Messier V, Rabasa-Lhoret R, Doucet E, Brochu M, Lavoie JM, et al (2010) Effects of the addition of a resistance training programme to a caloric restriction weight loss intervention on psychosocial factors in overweight and obese post-menopausal women: a Montreal Ottawa New Emerging Team study. J Sports Sci 28: 83–92. 20035493 10.1080/02640410903390105

[64] Nicklas BJ, Wang X, You T, Lyles MF, Demons J, et al (2009) Effect of exercise intensity on abdominal fat loss during calorie restriction in overweight and obese postmenopausal women: a randomized, controlled trial. Am J Clin Nutr 89: 1043–1052. 19211823 10.3945/ajcn.2008.26938 PMC2667455

[65] Straznicky NE, Lambert EA, Nestel PJ, McGrane MT, Dawood T, et al (2010) Sympathetic neural adaptation to hypocaloric diet with or without exercise training in obese metabolic syndrome subjects. Diabetes 59: 71–79. 19833893 10.2337/db09-0934 PMC2797947

[66] Wycherley TP, Noakes M, Clifton PM, Cleanthous X, Keogh JB, et al (2010) A high-protein diet with resistance exercise training improves weight loss and body composition in overweight and obese patients with type 2 diabetes. Diabetes Care 33: 969–976. 20150293 10.2337/dc09-1974 PMC2858200

[67] Toledo FGS, Menshikova EV, Azuma K, Radikova Z, Kelley CA, et al (2008) Mitochondrial capacity in skeletal muscle is not stimulated by weight loss despite increases in insulin action and decreases in intramyocellular lipid content. Diabetes 57: 987–994. 18252894 10.2337/db07-1429

[68] Hulens M, Vansant G, Claessens AL, Lysens R, Muls E (2003) Predictors of 6-minute walk test results in lean, obese and morbidly obese women. Scand J Med Sci Sports 13: 98–105. 12641641 10.1034/j.1600-0838.2003.10273.x

[69] Hulens M, Vansant G, Lysens R, Claessens AL, Muls E, et al (2001) Study of differences in peripheral muscle strength of lean versus obese women: an allometric approach. Int J Obes Relat Metab Disord 25: 676–681. 11360150 10.1038/sj.ijo.0801560

[70] Himes CL (2000) Obesity, disease, and functional limitation in later life. Demography 37: 73–82. 10748990

[71] Miller GD, Nicklas BJ, Davis C, Loeser RF, Lenchik L, et al (2006) Intensive weight loss program improves physical function in older obese adults with knee osteoarthritis. Obesity (Silver Spring) 14: 1219–1230. 16899803 10.1038/oby.2006.139

[72] Duvigneaud N, Matton L, Wijndaele K, Deriemaeker P, Lefevre J, et al (2008) Relationship of obesity with physical activity, aerobic fitness and muscle strength in Flemish adults. J Sports Med Phys Fitness 48: 201–210. 18427416

[73] Ruiz JR, Ortega FB, Castillo R, Martin-Matillas M, Kwak L, et al. (2010) Physical activity, fitness, weight status, and cognitive performance in adolescents. J Pediatr157: : 917–922 e911–915. 10.1016/j.jpeds.2010.06.026 20673915

[74] Vingard E, Blomkvist V, Rosenblad A, Lindberg P, Voss M, et al (2009) A physical fitness programme during paid working hours - impact on health and work ability among women working in the social service sector: a three year follow up study. Work 34: 339–344. 20037249 10.3233/WOR-2009-0932

[75] Genaidy AM, Karwowksi W, Guo L, Hidalgo J, Garbutt G (1992) Physical training: a tool for increasing work tolerance limits of employees engaged in manual handling tasks. Ergonomics 35: 1081–1102. 1505508 10.1080/00140139208967384

[76] Larrieu S, Peres K, Letenneur L, Berr C, Dartigues JF, et al (2004) Relationship between body mass index and different domains of disability in older persons: the 3C study. Int J Obes Relat Metab Disord 28: 1555–1560. 15314627 10.1038/sj.ijo.0802755

[77] Kraemer WJ, Mazzetti SA, Nindl BC, Gotshalk LA, Volek JS, et al (2001) Effect of resistance training on women's strength/power and occupational performances. Med Sci Sports Exerc 33: 1011–1025. 11404668 10.1097/00005768-200106000-00022

[78] Clemes SA, Haslam CO, Haslam RA (2010) What constitutes effective manual handling training? A systematic review. Occup Med (Lond) 60: 101–107. 19734238 10.1093/occmed/kqp127

[79] Craig BN, Congleton JJ, Kerk CJ, Amendola AA, Gaines WG (2006) Personal and non-occupational risk factors and occupational injury/illness. Am J Ind Med 49: 249–260. 16550563 10.1002/ajim.20290

[80] Allison DB, Zannolli R, Faith MS, Heo M, Pietrobelli A, et al (1999) Weight loss increases and fat loss decreases all-cause mortality rate: results from two independent cohort studies. Int J Obes Relat Metab Disord 23: 603–611. 10411233 10.1038/sj.ijo.0800875

[81] Webster JD, Hesp R, Garrow JS (1984) The composition of excess weight in obese women estimated by body density, total body water and total body potassium. Human nutrition Clinical nutrition 38: 299–306. 6469707

[82] Wycherley TP, Moran LJ, Clifton PM, Noakes M, Brinkworth GD (2012) Effects of energy-restricted high-protein, low-fat compared with standard-protein, low-fat diets: a meta-analysis of randomized controlled trials. The American Journal of Clinical Nutrition 96: 1281–1298. 23097268 10.3945/ajcn.112.044321

